# PPARs and Energy Metabolism Adaptation during Neurogenesis and Neuronal Maturation

**DOI:** 10.3390/ijms19071869

**Published:** 2018-06-26

**Authors:** Michele D’Angelo, Andrea Antonosante, Vanessa Castelli, Mariano Catanesi, NandhaKumar Moorthy, Dalila Iannotta, Annamaria Cimini, Elisabetta Benedetti

**Affiliations:** Department of Life, Health and Environmental Sciences, University of L’Aquila, 67100 L’Aquila, Italy; dangelomiche@gmail.com (M.D.); andrea.antonosante@gmail.com (A.A.); vanessa.castelli@graduate.univaq.it (V.C.); mariano.catanesi86@gmail.com (M.C.); nandhabinfo@gmail.com (N.M.); iannottadalila@gmail.com (D.I.); annamaria.cimini@univaq.it (A.C.)

**Keywords:** stem cells, metabolism, PPARs

## Abstract

Peroxisome proliferator activated receptors (PPARs) are a class of ligand-activated transcription factors, belonging to the superfamily of receptors for steroid and thyroid hormones, retinoids, and vitamin D. PPARs control the expression of several genes connected with carbohydrate and lipid metabolism, and it has been demonstrated that PPARs play important roles in determining neural stem cell (NSC) fate. Lipogenesis and aerobic glycolysis support the rapid proliferation during neurogenesis, and specific roles for PPARs in the control of different phases of neurogenesis have been demonstrated. Understanding the changes in metabolism during neuronal differentiation is important in the context of stem cell research, neurodegenerative diseases, and regenerative medicine. In this review, we will discuss pivotal evidence that supports the role of PPARs in energy metabolism alterations during neuronal maturation and neurodegenerative disorders.

## 1. Introduction

Neurogenesis, the process of generating neurons, occurs during embryonic and perinatal stages in mammals. It occurs also in the adult mammalian brain in two principal neurogenic niches, the subventricular zone (SVZ) of the lateral ventricles, and the subgranular zone (SGZ) of the dentate gyrus (DG) in the hippocampus [1]. Similarly to other adult stem cells, neural stem cells (NSCs) participate in tissue repair after brain damage. Consequently, it has been reported that neurogenesis follows different types of central nervous system (CNS) injury, including ischemic injury, seizure, and mechanical and excitotoxic injury. In line with the role of neurogenesis in the normal turnover of neuronal populations, recently through ^14^C, it has been demonstrated that about one third of the human adult hippocampal neurons is replaced with 700 new neurons per day [2]. Although, many transcription factors, participating in regulating adult neurogenesis, have been shown to control cell metabolism outside the brain [3]. Metabolism was, for a long time, considered to occur secondary to cell fate switch during neurogenesis. Nowadays, as recently reviewed by Lorenz and Prigione 2017, the emerging picture is that metabolism can be fine-tuned at different levels during neural commitment [4].

Glucose and lipid metabolism are regulated by transcriptional control exerted by peroxisome proliferator activated receptors (PPAR) α, β/δ, and γ, type II nuclear receptors that are particularly active in the brain [5]. In fact, PPAR isotypes are all expressed in the CNS (central nervous system) of rodents during embryonic development, as well as in adults. PPARβ/δ is broadly distributed in the brain, while PPARα and PPARγ are located in more restricted regions [6,7,8]. Although it has been demonstrated that PPARs can directly regulate neural cell differentiation [9,10,11,12,13,14] and play important roles in determining NSC fate [15,16,17,18]; less is known about their function in regulating NSC metabolism during differentiation. In this review, we will discuss some recent important evidence that supports the role of PPARs on adaptation of energy metabolism during neurogenesis, neuronal development, and neurodegenerative disorders. 

## 2. Metabolic States in Neural Stem Cells Lineage

NSCs are multipotent stem cells, which generate neurons and glial cells. NSCs use symmetrical division for a quick expansion of the progenitor pool; subsequently to the beginning of neurogenesis, they undergo an asymmetric division, by which a stem cell makes another stem cell and an intermediate progenitor committed to neurogenesis. The passage to gliogenesis involves a return to the symmetric division of progenitors [19]. During embryonic development, the choice between neuronal and glial fates is fine-regulated, particularly in vertebrates, in which different cell types are generated in a precise sequence: first neurons, followed by oligodendrocytes and astrocytes [20]. The specification of neuronal and glial cell types, consequently, may help to understand the complex interactions between multiple signaling pathways, transcription factors, and epigenetic mechanisms in the control of fate decision.

Metabolism can be fine-tuned at different levels during neural commitment, and it can play an important role in the specification of neuronal and glial cell types [4]. Neurons and glial cells have different metabolic programs; in fact, neurons are dependent on mitochondrial-based oxidative phosphorylation (OXPHOS), while glia stand on glycolysis [21,22]. NSCs, like glia cells, show a glycolytic nature, and this kind of metabolism is proposed to be an effect of cells’ elevated rate of proliferation, because it produces the precursor molecules for biomass generation via the pentose phosphate pathway (PPP) that results from the upstream branches of glycolysis [23]. In agreement with this concept, low oxygen typical of stem cell niches (<1–6%) [24] may influence cell metabolism, inducing anaerobic glycolysis. Hence, hypoxia induces stem cells self-renewal with respect to differentiation, and in concert, the hypoxia-inducible factors (HIFs) control the expression of genes involved in glycolysis and fructose metabolism [25]. Accordingly, in vivo evidence revealed that the modulation of blood vessel function in stem cell niches of the developing mouse cerebral cortex influenced neurogenesis in an oxygen-dependent manner [26]. The NSC state seems correlated with glycolytic metabolism coupled to non-fused mitochondrial morphology [27], while OXPHOS metabolism is commonly associated with differentiated neurons [22,28], which showed a typical tubular mitochondrial network. Recently, these concepts have been confirmed in several works investigating the mitochondrial state of neurons derived in vitro from human pluripotent stem cells (PSCs) [29,30,31]. Mitochondrial biogenesis and dynamics have a pivotal role in neuronal functions, since they regulate mitochondrial number, location, morphology, and function [32]. It is important to underline that these processes need synchronization refinement in the metabolic enzymes of fatty acid oxidation and oxidative phosphorylation [33], and PPARs are important regulators of these processes. Moreover, Mitofusin2 (Mfn2), a selective target of PPAR β/δ, [34], regulates mitochondrial fusion [35] and seems to be crucial for the efficiency of mitochondrial uptake of Ca^2+^ ions [36,37]. Although NSCs in vivo can rapidly divide during development, becoming quiescent in adult age [38], however, they still maintain glycolytic metabolism. One hypothesis to explain this behavior is that glycolytic metabolism also regulates redox metabolism; particularly, the use of glycolysis may reduce the intracellular levels of reactive oxygen species (ROS) [39]. Glycolysis produces reducing equivalents by means of the pentose cycle and, by reduced mitochondrial activity, promptly limits the generation of ROS. In fact, emerging evidence suggests that ROS can function as second messengers, playing a crucial role in the self-renewal of NSCs [40]. The correct intracellular ROS levels regulation may help to neurogenesis induction, suggesting that low ROS levels are beneficial for NSCs, while committed neural progenitor stem cells (NPCs) increase ROS production to promote differentiation [4]. However, also in NSCs, a determined amount of oxidative metabolism might even be necessary to prevent oncologic transformation of NSCs, as has been recently suggested that inhibition of mitochondrial metabolism in NSCs led to a switch towards more glycolysis with higher proliferation and less inducible differentiation [41]. A significant role in this control seems to be explained by de novo lipogenesis, in fact, an increase of fatty acid oxidation (FAO) was found to be high in adult NSCs in the SVZ, and pharmacological inhibition of FAO resulted in reduced proliferation [42]. In addition, de novo lipogenesis is crucial for adult stem cell behavior, as demonstrated by an interesting experiment of Knobloch et al., 2013, in which they showed a decrease of stem cell proliferation upon genetic deletion or pharmacological inhibition of the key enzyme fatty acid synthase [43]. Meanwhile, an elevated lipogenesis seems to be associated with an increase of NSC proliferation, and in quiescent NSCs, FAO appears, instead, to be favored. Data from single-cell RNA experiment demonstrate that a low rate oxidative metabolism, because of FAO in quiescent NSCs, may correspond to an alternative energy fuel to glucose [44]. Furthermore, congenital defects in mitochondrial FAO in NSCs, leads to differentiation with the loss of NSC self-renewal in the developing mouse brain [45]. In addition, silencing of promyelocytic leukemia gene (PML), which it is known to regulate FAO and is involved in modulation of PPAR β/δ signaling, reduces the hematopoietic stem cell pool in mice [46]. 

In the brain, during pathological conditions, an alteration in metabolic status occurs; in fact, recent studies showed an impaired NSCs function in metabolic disease underlying the role of lipid metabolism in neurogenesis. In example, high fat diet (HFD) decreases hippocampal neurogenesis in male rats. These mice exhibit reduced hippocampal neurogenesis and neuronal precursor cells proliferation paralleled with increased lipid peroxidation and decreased expression of trophic and pro-neurogenic BDNF (brain derived neurotrophic factor). Moreover, young mice treated with HFD exhibited decreased hippocampal neurogenesis respect adult mice under the same diet [2]. It has been demonstrated that lipid accumulation perturbs niche microenvironment and inhibits neurogenesis in unhealthy brains, thus supporting evidence for a novel FA-mediated mechanism suppressing NSC activity. 

In this context, it is important to underline recent evidence suggesting that sporadic Alzheimer’s disease (AD) etiopathogenesis could also involve dysfunctional brain insulin signaling, with subsequent glucose dysmetabolism and metabolic shift to alternative energy sources, also known as type 3 diabetes [47]. 

## 3. Roles of PPARs in the Energetic Metabolic Switch Occurring during Neurogenesis and Neuronal Maturation

PPARs are ligand-activated transcription factors included into nuclear receptor superfamily, three isotypes have been determined, encoded by separate genes (α, *NR1C1*; β/δ, *NR1C2*; and γ, *NR1C3*). PPARs, once activated by the ligand, form a heterodimer with the 9-cis retinoic acid receptor (RXR) and modulate the transcription of their target genes by binding to the putative PPRE (AGGTCAAAGGTCA) in the promoter regions of them. Regarding their protein structure, in the N-terminal there is the A/B domain (AF-1), which holds a ligand-independent function, while the C-terminal domain, that holds the DNA binding domain (DBD), is composed of two zinc finger-like motifs that can bind the PPARs response element (PPRE). The D domain is a hinge region important for the cofactor interaction, and consequently, for DNA binding. The E/F (LBD) domain is involved in the dimerization with RXR and a ligand-dependent transcriptional activating function (AF-2) [38,48]. PPARs transcriptional activity and stability can be modified covalently by phosphorylation, ubiquitylation, and SUMOylation [49,50]. PPARα, the first PPAR to be identified, is expressed mainly in the liver, heart, and brown adipose tissue, in which it regulates the ketogenesis, lipid storage, and fatty acid oxidation pathways. PPARβ/δ is ubiquitously expressed, and it has a leading role in glucose and fatty acid oxidation in key metabolic tissues, such as liver, skeletal muscle, and heart. Finally, PPARγ is expressed in white adipose tissue, where it is a master regulator of adipogenesis, as well as a potent modulator of whole-body lipid metabolism and insulin sensitivity [51]. 

Regarding PPAR ligands, some of them, such as fibrates (PPARα ligands), are currently used as treatment of dyslipidemia; while, glitazones (PPARγ ligands) are antidiabetic and insulin-sensitizing agents, otherwise, PPARβ/δ ligands have only confirmations obtained from animal models [52]. Moreover, PPARα/γ dual agonists, (glitazar) PPAR α/δ dual agonists (elafibranor), and pan-PPAR agonists have been recently become available [52]. 

Regarding their expression in the brain, all PPAR isotypes are expressed in CNS, both during embryonic development and in the adult. PPARα and PPARγ are located in more restricted regions, while PPARβ/δ is widely distributed in the brain [6,7,8]. PPARs are implicated in the regulation of the proliferation, migration, and differentiation of NSCs by signaling pathways, such as STAT3, NFkB, and Wnt [15,16,17], and it has been demonstrated that in neurospheres, grown in vitro from adult mouse SVZ, all three PPAR isotypes are expressed [18,53]. PPARβ/δ resulted the most abundant isotype; it is not surprising due to its early expression and its abundance during brain development [6]. Moreover, the concurrent expression of the three isotypes in the NSC nucleus does not mean that they are all transcriptionally active; in fact, it has been suggested that unliganded PPARβ/δ may act as potent inhibitor of the transcriptional activity of α and γ isotypes [54]. In the astroglial differentiating NSCs, PPARs undergo quantitative modifications. A strong decrease of PPARβ/δ was observed, in this context, it might be considered as inhibitor of astroglial differentiation. PPAR*γ* did not change, both at mRNA and protein levels, while PPARα was significantly increased in agreement with our previous findings on astrocytes in vitro differentiation [14], suggesting a role for this transcription factor in astroglial differentiation, confirmed by the results achieved when NSCs were treated with a specific PPARα agonist [18]. Finally, in the cytoplasm of neural stem cells, large lipid droplets were found in SVZ adult NSCs, in accordance with de novo lipogenesis [42]. Moreover, lipid droplet withdrawal, during astroglial differentiation, agrees with the view that differentiated astrocytes develop catabolic lipid metabolism, rather than anabolic, needing PPARα activity. 

**Figure 1:**
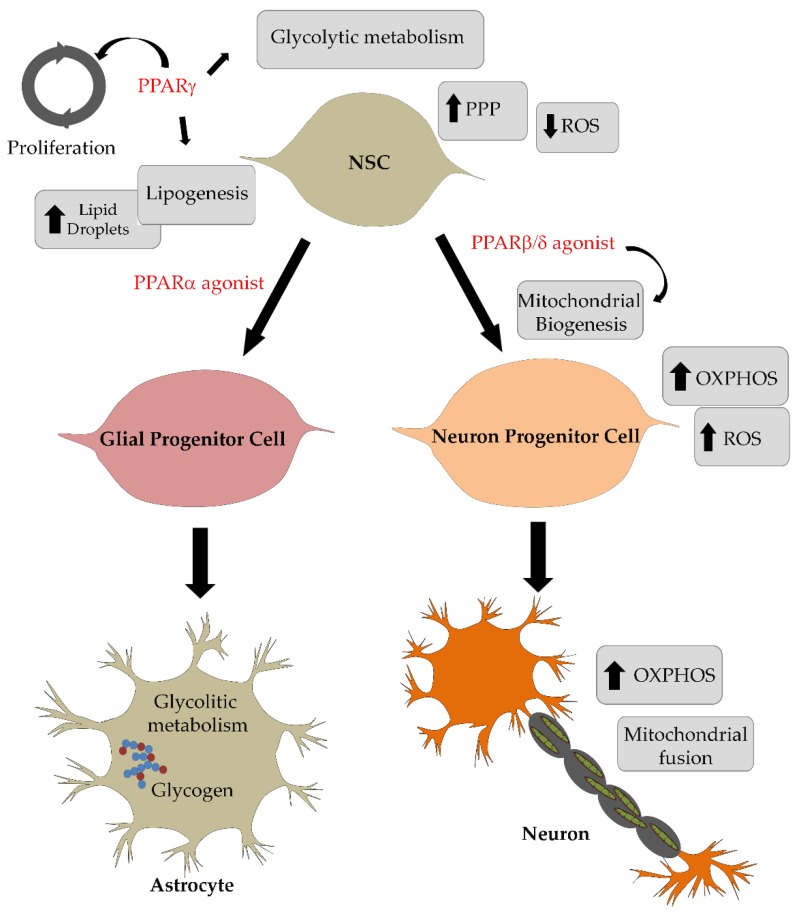
Scheme summarizing the effects of peroxisome proliferator activated receptors (PPARs) on energy metabolism adaptation during neural stem cells differentiation in neurons and astrocytes.

In Figure 1, is shown a scheme summarizing the effects of PPARs on energy metabolism adaptation during neural stem cell differentiation in neurons and astrocytes. 

## 4. Roles of PPARβ/δ in Neurogenesis and Neuronal Maturation

The PPARβ/δ isotype is highly expressed in the brain [55], and its deletion in mice is associated with brain developmental defects [56]. In fact, PPARβ/δ has important roles in neuronal function; it has been demonstrated that PPARβ/δ-deficient mice are viable, but they show several defects in CNS such as altered myelination [56] and bad performance in memory tests, paralleled with an increase in inflammatory markers, astrogliosis, and tau hyperphosphorylation [57]. The presence and modulation of PPARβ/δ in embryonic rat cortical neurons during their in vitro maturation were observed by us [9], suggesting a potential role of PPARβ/δ in neuronal maturation. In addition, we demonstrated in human neuroblastoma cell line, SH-SY5Y, a neuronal differentiating effect of PPARβ/δ [58,59]. The signal transduction pathways activated by PPARβ/δ during neuronal differentiation were studied on this in vitro model. In particular, it has been demonstrated that the PPARβ/δ activation was able to determine the activation of MAPK-ERK1/2 and to increase the expression of BDNF and p75 receptor, in parallel to a decrease in BDNF TrkB receptor, suggesting that activation of PPARβ/δ was involved, directly or indirectly in neuritogenesis and neuronal maturation. Finally, these results were further confirmed by the use of a specific agonist and antagonist of PPAR β/δ in primary neuronal cultures [11], in which we also observed a specific effect of PPARβ/δ activation on cholesterol biosynthesis during neuronal maturation. Furthermore, it has been demonstrated that retinoic acid (RA) promotes neurogenesis by activating both retinoic acid receptors (RARs) and PPAR β/δ in P19 mouse embryonal carcinoma cell line [10]. Recently, Mei and Coll, in 2016, have been reported that, by modulating mitochondrial energy metabolism via Mfn2 and mitochondrial Ca^2+^, PPAR β/δ plays a key role in neuronal differentiation. This study provides novel insights for the role of PPARβ/δ and energy metabolism adaptation during neurogenesis and neuronal maturation [33]. In particular, the authors have been shown that flavonoid compound 4a facilitated embryonic stem cells (ESC) to differentiate into neurons morphologically as well as functionally, and that the PPAR β/δ gene silencing blocked compound 4a-induced neurogenesis of ES cells, demonstrating the important role of PPARβ/δ in neuronal differentiation. In this kind of model, mitochondrial biogenesis was upregulated by compound 4a treatment, and was altered by sh-PPAR β/δ knockdown, suggesting a key role of PPAR β/δ in mitochondrial biogenesis during neuronal differentiation. Moreover, they showed that the compound 4a was able to increase the protein expression of Mfn2, which was abolished by PPARβ/δ knockdown, and that sh-PPAR β/δ reduced mitochondrial Ca^2+^ concentration. Thus, PPARβ/δ seems strongly implicated in the induction of neuronal lineage, increasing mitochondrial fusion, modulating BDNF expression, cholesterol biosynthesis, and mitochondrial FAO. Finally, it should be emphasized that a natural ligand of this receptor, the 4-hydroxynonenal (4-HNE) [60], is a product of oxidative stress and, thus, it should be possible that the increased ROS levels in committed neuroblast could trigger the activation of PPAR β/δ. 

## 5. Roles of PPARγ in Neurogenesis and Neuronal Maturation

PPARγ activation induces the transcription of genes associated with lipid uptake and storage, playing critical roles in lipid homeostasis [61]. PPARγ controls murine NSC proliferation and survival [27]; particularly, when activated by low concentrations of specific agonists, PPARγ stimulates proliferation concurrently constraining neuronal differentiation, while activation by high concentrations of agonists leads to NSC death. This dual role suggests that PPARγ controls the expansion of NSC population in a concentration-dependent manner, and it shows that precise concentrations of its agonists are critical for the survival and proliferation of NSCs in vivo. 

Regarding metabolism, in order to examine the mechanisms of PPARγ in the control of energy balance in CNS, Stump and colleagues 2016 used a Cre-recombinase dependent (Nestin^Cre^), conditionally activatable transgene expressing either wildtype (WT) or dominant-negative (P467L) PPARγ. What they found is that Nes^Cre^/PPARγ-WT mice displayed severe microcephaly and brain malformation, indicating that PPARγ can control brain development. On the contrary, global interference with PPARγ function caused impaired growth, resistance to diet induced obesity, decreased lean mass, redistribution of adipose tissue, GH resistance, and abnormalities in glucose and insulin [62]. 

Recently, we have shown, in vitro, the energetic metabolism pathways controlled by PPARγ [63] in neuroblast differentiation. We used the human neuroblastoma cell lines SH-SY5Y, as a model of neuroblast induced to differentiate neuron. During the early phases of neuronal differentiation, a significant downregulation of PPARγ was observed, concomitant with a change in its cellular localization, in fact, it came to be cytoplasmic after the differentiation challenge. In addition, we observed that the decrease of PPARγ was paralleled by a strong decrease of glycogen and lipid droplets content in differentiating cells. PPARγ knockdown showed a strong decrease of glycogen content, concomitant with a significant increase of phosphorylase glycogen brain (PYGB), indicating that PPARγ is critical for NPCs maintenance and energetic storage.

## 6. Energy Metabolism Imbalance in Neurodegenerative Disorders

During aging, there is an increase of circulating glucose due to the cellular inability to increase glucose uptake in response to insulin, and this peripheral insulin resistance has been related with poorer cognitive function [64]. Insulin signaling pathway results in phosphorylation of the insulin receptor-interacting protein (IRS-1), particularly, a decrease in IRS-1 phosphorylation may induce insulin resistance, while an increased phosphorylation on serine 312 of IRS-1 has opposite effects. Studies on post mortem brain tissue from elderly subjects showed an increased IRS-1 phosphorylation on serine 312, suggesting neuronal insulin resistance [65,66]. Concomitant with insulin resistance, also, the neuronal glucose transporter GLUT3 is susceptible to aging factors [67,68]. During aging, the metabolism of several lipid species is altered, such as long-chain ceramides [69] and omega-3 fatty acids [70]. Dyslipidemia is often associated with dementia, and it may increase the risk of AD [71]. Moreover, individuals having the ε4 allele of the gene encoding apolipoprotein E, the protein that transports cholesterol and lipoproteins, have an increased risk of developing sporadic AD [72].

Accordingly, age-related neurodegenerative disorders, such as AD and PD, share common pathogenic pathway with metabolic syndromes like obesity and type 2 diabetes, such as deregulation of brain insulin signaling and insulin growth factor-1 (IGF-1) signaling. This signaling induces insulin resistance, and energy and lipid metabolism imbalance, that have a direct negative impact on the CNS [47]. Moreover, neurodegenerative disorders, such as metabolic syndromes, are characterized also by mitochondrial and peroxisomal dysfunction, and alterations in energy metabolism [73,74].

Alzheimer’s disease is the most common form of dementia, characterized by age-related cognitive decline that starts as mild short-term memory impairment, and then progresses to severe deficits in essentially all cognitive domains. The hallmarks of this disease are amyloid β plaques (Aβ) and hyperphosphorylated tau tangles [75]. 

Parkinson’s disease (PD), like AD, is a long-term degenerative disorder of the CNS, characterized by degeneration of dopaminergic neurons in the substantia nigra that innervate the striatum [76]. The hallmarks of PD are “Lewy bodies”, large accumulations of α-synuclein in the cytoplasm [77]; experimental evidence suggests that the accumulation of α-synuclein aggregates induces mitochondrial dysfunction in neurons, and these are pivotal events in the pathogenesis of PD.

As reviewed by Agarwal and colleagues 2017, it is becoming increasingly evident that mitochondrial abnormalities play an import role in the onset, progression, and neuronal cell death in age-related neurodegenerative disorders [73]. 

Recently, in neurodegenerative disorders, it has been demonstrated that functional and structural changes in mitochondria are early features that conduce to neuronal death, paralleled by cognitive and neurobehavioral abnormalities [78]. In age-related neurodegenerative disorders, the mitochondrial population is decreased, due to dysregulation of mitochondrial biogenesis [79]. The mitochondrial dysfunction observed in neurodegenerative disorders leads to the damage in mitochondrial electron transport chain, in the mitochondrial DNA, and calcium buffering [79]. Mitochondria is the second major intracellular Ca^2+^ store after endoplasmic reticulum, and Ca^2+^ deregulation plays a critical role in the pathogenesis of several neurodegenerative disorders [80]. In fact, mitochondrial Ca^2+^ plays an important role in preserving cellular physiology, activating the respiratory chain [81]. When mitochondria accumulate excessive Ca^2+^ ions, this causes mitochondrial swelling, injury of mitochondrial membrane potential, and finally, it induces apoptosis in neurons [82]. 

Mitochondrial dynamics/biogenesis helps to maintain the characteristic morphology of mitochondria and a healthy mitochondrial pool in neurons; it is a tightly controlled balance between three important phenomena: mitochondria fission, fusion, and degradation. [78]. Mitochondrial fission consists of replacement of damaged mitochondria, and it plays a main role in the appropriate function and assembly of mitochondrial electron transport chain complex [78]; the main protein mediators of mitochondrial fission are Fis-1 and Drp-1 [78]. Fusion is related with the improvement of mitochondrial functions, and is regulated by three main proteins: mitofusin 1 (Mfn-1), mitofusin 2 (Mfn-2), and optic atrophy protein 1 (OPA-1) [78]. The expression and protein levels of Drp-1, Opa-1, Mfn-1, and Mfn-2 are decreased in numerous neurodegenerative disorders. Moreover, mutations in several PD-linked genes, like *PINK-1*, *Parkin*, *DJ-1*, *LRRK2*, and *VPS35*, are directly or indirectly, linked to mitochondrial dysfunction [83,84]. In particular, PINK/parkin pathway promotes mitochondrial fission or inhibits mitochondrial fusion in drosophila [85]. A key factor for mitochondria biogenesis is the PGC-1 α; any loss or impairment in PGC-1α activity may result in metabolic defects and mitochondrial dysfunctions in most neurodegenerative disease [78]. PPARs bind this transcriptional co-activator, modulating the expression of the gene encoding for mitochondrial fatty acid oxidation and glucose metabolism enzymes [86], but also the genes encoding for antioxidant enzymes such as catalase, glutathione peroxidase, and MnSOD, thus reducing oxidative damage [87,88]. 

The role of peroxisomal dysfunction in aging has been largely undervalued; however, accumulating evidence suggests that peroxisomal function declines with aging and in age-related neurological disorders, such as AD and PD [89]. Interestingly, not only mitochondria, but also peroxisomes, are organelles involved in the response to the redox unbalance, characterizing the earliest phases of Aβ pathology [90,91,92].

Peroxisomal dysfunction was also linked to disease, principally through ROS metabolism [93,94], in fact, peroxisome-mediated ROS production may have also a deeper effect on mitochondrial integrity, as demonstrated by the induction of intraperoxisomal ROS, using a peroxisome-localized photosensitizer [95]. Interestingly, genetic inactivation of catalase, a PPAR target gene, perturbs mitochondrial redox potential in mice [96]. Reflecting the intimate link between the two organelles, these studies suggest that peroxisomal dysfunction may be a precursor for mitochondrial impairment. Moreover, proteins involved in peroxisomal fatty acid oxidation, ether lipid synthesis, and other peroxisomal processes, were also decreased in in age-related neurological disorders [93], suggesting that peroxisomal dysfunction extends beyond dysregulated ROS metabolism. Remarkably, increased very long chain fatty acids (VLCFAs) and reduced plasmalogen levels are observed in the brain of AD patients, suggesting a possible defect in peroxisomal beta oxidation and peroxisomal lipid synthesis [97]. Peroxisomal dysfunction is present also in PD, particularly, plasmalogen levels are significantly reduced in PD post mortem human frontal cortex lipid rafts [98].

## 7. Roles of PPARs in Neurodegenerative Disorders

The most studied PPAR in neurodegenerative disease is the γ isotype. Combs and colleagues [99] were the first to report the relationship between PPARγ activation and neurodegeneration, and this evidence was supported by several lines of evidence in animal and cellular models of Alzheimer’s disease (AD), Parkinson’s disease (PD), amyotrophic lateral sclerosis (ALS), Huntington’s disease (HD), stroke, and traumatic injuries [100]. 

In numerous mouse models of AD, it has been indicated that administration of PPARγ agonists can ameliorate memory and cognition performance, reduce inflammation, and decrease amyloid levels. Searcy and colleagues [101] have been demonstrated that PPAR agonists are able to ameliorate synaptic function in AD mouse models. 

Since it is known that PPARγ agonists decrease insulin resistance in type II diabetes, the beneficial effects of PPARγ agonists in AD mice indicate that they can act in the same manner in CNS [102]. Escribano and his research group demonstrated that rosiglitazone, a high-affinity PPARγ agonist, rescues memory impairment in a mouse model of AD [103]. Specifically, these authors indicated that rosiglitazone promotes Aβ clearance, by promoting microglial phagocytic ability and decreasing the expression of proinflammatory markers. 

Moreover, an interesting meta-analysis compared the efficacy of glitazones (antidiabetic and insulin-sensitizing agents) for Alzheimer’s disease (AD) and mild cognitive impairment (MCI). In particular, this analysis included 20 comparisons from 4855 individuals randomly assigned to 6 different antidiabetic drugs with various doses. The results have shown that pioglitazone and rosiglitazone had the major pro-cognitive effects in subjects with AD/MCI [104].

Recently a role for PPARγ has been recognized in regional transcriptional regulation of chr19q13.32; this region contains genes such as *TOMM40* and *APOE*, implicated in AD. Mostly, this region holds a number of PPARγ binding sites, and understanding how those sites regulate the expression of genes in the region could help in the development of more efficient therapies [105].

In a recent study, Cheng and collaborators (2015) studied the effects of PPARα activation on neuronal degeneration by inducing Aβ42 cytotoxicity in an in vitro model. They established that the mitochondrial-associated AIF/Endo G-dependent pathway could be prevented by activation of the receptor in this model [106]. Recently, Fidaleo et al. [107] reported that PPARα ligands, such as palmitoylethanolamide (PEA), are able to protect neurons from degeneration, leading to a reduction in oxidative stress, inflammation, and neurogenesis, and glial cell proliferation/differentiation, thus further suggesting the use of PPARα as a potential therapeutic agent for neurodegeneration.

In 2003, Brune and colleagues [108] screened for polymorphisms in the PPARα gene, and they detected two known polymorphisms located in exon 5 and intron 7. They studied the possible association of these polymorphisms with AD and its effect in carriers of an insulin gene (*INS*) polymorphism. They showed that carriers of a *PPARαL162V* allele and an *INS-1* allele presented an increased risk for AD. These authors also found an increased level of βamyloid in cerebrospinal fluid in PPAR-α L162V genotype carriers. These results suggested that PPARα polymorphism may be considered a risk factor for AD. Moreover, since altered glucose metabolism has been indicated in AD, the interaction of the insulin and the PPARα genes in AD risk in the Epistasis Project, have been assayed. The authors proposed that dysregulation of glucose metabolism leads to the development of AD, and might be due, in part, to genetic variations in *INS* and *PPARα*, and their interaction especially in Northern Europeans [109]. Recently, it has been reported that statins serve as ligands of PPARα, and that Leu331 and Tyr 334 residues of PPARα are important for statin binding [110]. Upon binding, statins induce upregulation of neurotrophins through PPARα-mediated transcriptional activation of cAMP-response element binding protein (CREB). Consequently, simvastatin increases CREB and also BDNF in the hippocampus of PPARα null mice receiving full-length lentiviral PPARα, but not L331M/Y334D statin-binding domain mutated lentiviral PPARα. This study identifies statins as ligands of PPARα analyzing the importance of PPARα in the therapeutic success of simvastatin in an animal model of Alzheimer’s disease. Limited studies indicated a protective role for PPARα agonists in models of PD: treatment with the PPARα agonist fenofibrate [111] protected nigral dopaminergic neurons in the 1-methyl-4-phenyl-1,2,3,6-tetrahydropyridine (MPTP) mouse model of PD. The role of PPARβ/δ in neurodegeneration is less studied than PPARγ and α, and more controversial. PPARβ/δ agonists, acting through PPARβ/δ activation, induce protection in many pathological CNS states, such as a transgenic mouse model of Alzheimer’s disease, MPTP model of Parkinson’s disease, stroke, EAE, spinal cord injury and in a streptozotocin-induced experimental type 3 diabetes [100]; in all these cases, the effect has been mainly attributed to reduction of inflammation and oxidative stress. However, the main question regarding this nuclear receptor is that further studies are needed in order to better characterize this receptor in a more systemic manner, to support the possibility that PPARβ/δ might be used as a therapeutic target [112].

Regarding mitochondrial biogenesis, PPAR agonists can increase the functionality of mitochondrial, and they enhance Ca^2+^ buffering ability of mitochondria. Therefore, it seems attractive to examine the cellular and molecular mechanisms by which PPARs determine changes in cytosolic Ca^2+^ concentration to develop new strategies in the field of drug development for neurodegenerative disorders [73]. Moreover, PPAR agonists are able to induce mitochondrial biogenesis through PGC-1α, preventing mitochondrial dysfunction caused by oxidative insults [113]. In Table 1, are shown the references on energy metabolism imbalance in neurodegenerative disorders, and about PPARs ligands. 

## 8. Conclusions

The data summarized here underlines the significant role of PPARs in energy metabolism adaptation during brain development. However, we still need to better elucidate the molecular networks driven by these nuclear receptors in regulating NSC metabolism during self-renewal and differentiation. In the brain, during pathological conditions, an alteration in metabolic status occurs, whereby elucidate the crucial steps in energetic metabolism and the involvement of PPARs in NSCs neuronal fate (lineage) may be useful for the future design of preventive and/or therapeutic interventions. However, the future use of PPAR ligands as therapeutic agent is related to an important problem of design of drugs: the new molecules have to be able to pass the BBB (blood–brain barrier) and they have to be projected in order to avoid the classical pharmacokinetic problems related to the drugs active on CNS.

## Figures and Tables

**Table 1:** Table summarizing the references on energy metabolism imbalance in neurodegenerative disorders and on PPARs and PPAR ligands.

Neurodegenerative Diseases i.e.		
AD	and	PD
Ref. Energy Metabolism Imbalance		Ref. PPARs and Their Ligands
[47,64,65,66,67,68]	Insulin Resistance	[102,103,104,109,110]
[78,79,80,81,82,83,84,85]	Mitochondrial Dysregulation	[73,105,106,113]
[89,90,91,92,93,94,95,96,97,98]	Peroxisomal Dysregulation	[90,91,92]
